# Exosomes Containing LINC00636 Inhibit MAPK1 through the miR-450a-2-3p Overexpression in Human Pericardial Fluid and Improve Cardiac Fibrosis in Patients with Atrial Fibrillation

**DOI:** 10.1155/2021/9960241

**Published:** 2021-06-28

**Authors:** Langsha Liu, Fanyan Luo, Kaibo Lei

**Affiliations:** Department of Cardiac Surgery, Xiangya Hospital, Central South University, Changsha, China

## Abstract

The purpose of this study was to investigate the regulatory mechanism of miR-450a-2-3p in myocardial fibrosis in patients with atrial fibrillation. For this purpose, the expression profile of GSE55296 was extracted from the GEO database, and differentially expressed lncRNAs were identified. Gene ontology analysis of the target genes of mir-450a-2-3p indicated that there was a regulatory relationship between LINC00636 and miR-450a-2-3p. Further, the expression levels of the analyzed RNAs were confirmed by RT-qPCR. TGF-*β*1-induced cardiac fibroblasts (CFs) and human umbilical vein endothelial cells (HUVECs) were used to establish a myocardial fibrosis model and endothelium-mesenchymal transformation (EMT) model in vivo. We hypothesized that exosomes containing LINC00636 regulate the expression of miR-450a-2-3p. LINC00636 was positively correlated with the expression of miR-450a-2-3p. The overexpression of miR-450a-2-3p suppressed the MAPK1 expression in CFs, thereby inhibiting the expression of *α*-SMA, COL1, and COL3 and preventing CF proliferation. In HUVECs, the miR-450a-2-3p overexpression upregulated the expression of VE-Cadherin (VE-Cad) and platelet endothelial cell adhesion molecule-1 (PECAM-1/CD31) by inhibiting the mitogen-activated protein kinase 1 (MAPK1) expression, whereas the expression levels of vimentin, COL1, and COL3 decreased. These results indicate that LINC00636, which is present in human pericardial fluid, is an antifibrotic molecule that inhibits MAPK1 through the miR-450a-2-3p overexpression and improves cardiac fibrosis in patients with atrial fibrillation.

## 1. Introduction

Atrial fibrillation (AF) is one of the most common sustained supraventricular arrhythmias that increase the risk of stroke [1]. The underlying mechanism is thought to be associated with electrical and structural atrial remodeling, which lead to cardiac fibrosis [2, 3]. The activation and proliferation of cardiac fibroblasts (CFs), which produce excessive extracellular matrix (ECM) components, such as collagen-1 (COL1) and collagen-3 (COL3), play a pivotal role in fibrogenesis in patients with AF [4]. Furthermore, CFs can differentiate into myofibroblasts, the cells that present a 2-fold higher ability to synthesize collagen, including alpha smooth muscle actin (*α*-SMA) [5]. Although in physiological conditions cardiac fibrosis is low, differentiation of monocytes, endothelial cells, bone marrow circulating progenitor cells, and peripheral cells in pathological conditions increase cardiac fibrosis [6–8]. Consequently, endothelial-mesenchymal transition (EMT) in endothelial cells is closely related to cardiac fibrosis. However, the molecular mechanisms of cardiac fibrosis and EMT in AF are largely unclear.

Long noncoding RNAs (lncRNAs) are a subclass of transcripts longer than 200 bp without identifiable open reading frames or protein-coding capacity that are derived from intergenic regions (the antisense strand of a given gene) of the genome, introns of protein-coding genes, or through alternative splicing [9]. Recent research has shown that the lncRNA LINC00636 affects the proliferation, apoptosis, migration, and invasion of cervical cancer cells [10]. However, the influence of LINC00636 on CFs has not yet been studied. Our previous comprehensive bioinformatic analysis validated with RT-qPCR showed that miR-450a-2-3p was downregulated in CFs exposed to transforming growth factor (TGF)-*β*1 and human umbilical vein endothelial cells (HUVECs) and may act as microRNAs (miRNAs) that inhibit fibrosis [11]. miRNAs are capable of regulating the gene expression; they reduce the gene expression by degrading or functionally inhibiting target mRNAs after transcription [12]. MiRNAs produced from parental cells in bioactive forms are not destroyed in biological fluids, thereby exhibiting great potential as disease biomarkers [13]. Hence, the components of the pericardial fluid (PF) that surround the heart, epicardial adipose tissues, and large vascular roots that carry blood to the heart may reflect the myocardial protein expression profile. Recent research has shown that miRNAs can promote angiogenesis of endothelial cells through exosomes in human PF [14]. However, their role in the atrial remodeling of exosomes in AF is not well characterized.

Bioinformatic analysis suggested that the miR-450a-2-3p/MAPK1 pathway affects cardiac fibrosis. Based on this result, we focused on upstream target molecules of the miR-450a-2-3p/MAPK1 pathway and performed functional phenotype verification at the cellular level. Specifically, we focused on the effects of LINC00636 downregulation in CFs and hypothesized that LINC00636 mediates miR-450a-2-3p to affect the CF cellular activity. Furthermore, we demonstrated that MAPK1 acts as a downstream target of miR-450a-2-3p and functions as a fibrogenic gene in fibrosis progression. Collectively, the goal of this study was to discover and identify a cardiac fibrosis-associated lncRNA and its molecular mechanism using bioinformatic analyses. Our findings may help identify the possible mechanisms of AF and contribute to the development of more promising therapeutic targets.

## 2. Material and Methods

### 2.1. Collection and Treatment of PF Samples

The study was conducted in accordance with the Declaration of Helsinki, as revised in 2013. All protocols were approved by the Ethics Committee of Xiangya Hospital Central South University (201803209), and informed consent was obtained from all participants. We isolated exosomes present in the PF of nAF for subsequent experiments. In addition, total RNA was extracted from the PF exosomes of patients with nAF (*n* = 12) or AF (*n* = 12).

### 2.2. Bioinformatic Analyses

For our study, we selected Gene Expression Omnibus (GEO, http://www.ncbi.nlm.nih.gov/geo), which is a publicly accessible database containing gene/microarray profiles. The gene expression profile GSE55296 was extracted from the GEO database. Using software *R* and the ggplot2 (3.3.0) package, we generated volcano plots and boxplots of the selected gene expression profile. The lncRNA-to-miRNA prediction was performed using lncBase (http://carolina.imis.athena-innovation.gr/diana_tools/web/index.php?r =lncbasev). Using the igraph package in *R*, we created a network map. Finally, we created an enrichment map using the cluster Profiler_3.11.0 package in *R*.

### 2.3. Isolation and Extraction of Exosomes from PF

PF exosomes were isolated using THE ExoQuick kit (System Biosciences, PA, USA) according to manufacturer's instructions. Briefly, after centrifugation of PF at 3,000 × *g* for 15 min, the pellet containing cell debris was discarded, and the supernatant was transferred to a sterile container. An appropriate volume of ExoQuick-exosome precipitate solution was added to the PF. The ExoQuick/PF mixture was centrifuged again at 1,500 × *g* for 30 min, and the supernatant was removed using suction. The remaining pellet, containing exosomes, was resuspended in PBS.

### 2.4. Transmission Electron Microscopy (TEM)

Phosphotungstic acid (approximately 1%) was prepared in a phosphate buffer. The suspension sample (10 *μ*l) was dropped onto a coated copper mesh, which was subsequently incubated in an oven at 37°C. A filter paper cut into shapes with sharp corners was used to absorb the staining solution. Thereafter, phosphotungstic acid staining solution (10 *μ*l) was dropped on the copper mesh with the prepared sample using sample gun. After 1–2 min, excess phosphotungstic acid was washed off. Transmission electron microscopy (TEM) was performed using a HT-7800 microscope (Hitachi, Japan) to observe and capture images of the exosome morphology.

### 2.5. Fluorescent Imaging of Exosome Uptake

A total of 50 *μ*L of sample containing exosomes isolated from the human PF were diluted with Diluent C to a final volume of 250 *μ*L and labeled with 1.5 *μ*L of PKH26 dye. Then, 150 *μ*L of exosome samples wAS incubated with CFs or HUVECs overnight at 4°C. After washing, the cells were stained with 1 ml of 1 *μ*g/ml DAPI, and pictures were captured using a Motic inverted fluorescence microscope.

### 2.6. Cell Culture and Treatment

Both HUVECs and rat primary CFs used in this study were purchased from ScienCell (CA, USA). After 3–8 generations, the cells were selected for experimental verification. HUVECs were cultured in Dulbecco's Modified Eagle's Medium (DMEM) containing 10% fetal bovine serum (FBS) and 1% penicillin-streptomycin. CF cultures were supplemented with 1% fibroblast growth supplement-2 (ScienCell, CA, USA).

The cells were digested with 0.25% trypsin (Beyotime Biotechnology, Shanghai, China) (containing 0.02% EDTA) at 37°C for 5 min followed by centrifugation at 1,000 rpm and then seeded into 6-well plates. A total of 20 *μ*L of Lipo2000 (Invitrogen, CA, USA) diluted in 1 ml of serum-free DMEM was mixed with 10 *μ*L each of mimic, mimic-control, inhibitor, and inhibitor-control (Honorgene, Changsha, China) diluted in 250 *μ*L of serum-free DMEM for 20 min. The transfection medium was replaced with DMEM containing FBS after 6 h. After 24 h, the cells were incubated with 50 ng/ml of recombinant human TGF-*β*1 for 24 h.

### 2.7. RNA Extraction and Quantitative Real-Time Analysis (RT-qPCR)

Total RNA was extracted according to the manufacturer's instructions. Briefly, 1 ml of TRIzol (Thermo Fisher Scientific, MA, USA) was added per 10 cm^2^ of the plate surface area. mRNAs were reverse transcribed using a reverse transcription kit (Cowin, Beijing, China). RT-qPCR was performed using an UltraSYBR mixture (Cowin, Beijing, China). The relative expression of genes was calculated using the 2^−*ΔΔ*CT^. The miRNA expression of samples was normalized with that of U6 small nuclear RNA, and the mRNA expression of samples was normalized with that of *β*-actin. All primers used in this study were commercially obtained from Sangon (Shanghai, China) and are shown in Supplementary Table 1.

### 2.8. Western Blot Analysis of Protein Expression Levels

Cells or exosomes were lysed in RIPA buffer (Beyotime Biotechnology, Shanghai, China). After centrifugation at 12,000 × *g* for 15 min at 4°C, the supernatant was collected. Protein concentration was determined using the bicinchoninic acid assay using a commercial kit (Cwbiotec, Beijing, China). Next, 160 *μ*L of protein was separated using a 40 *μ*L 5× loading buffer and then transferred to a nitrocellulose filter membrane. The membrane was incubated in 5% bovine serum albumin (BSA) at 4°C for 1.5 h and incubated overnight with primary antibody at 4°C. The primary antibodies used for western blotting were as follows: *β*-actin (1 : 5000; Proteintech, IL, USA), CD9 (1 : 2,000; Proteintech, IL, USA), CD63 (1 : 1000; Proteintech, IL, USA), Tsg101 (1 : 5000; Abcam, Cambridge, United Kingdom), *α*-SMA (1 : 1000; Proteintech, IL, USA), COL1 (1 : 1000; Bioss, Beijing, China), COL3 (1 : 500; Proteintech, IL, USA), MAPK1 (1 : 1000; Proteintech, IL, USA), VE-Cad (1 : 1000; CST, MA, USA), PECAM-1/CD31 (1 : 2000; Proteintech, IL, USA), and vimentin (1 : 2000, Proteintech, IL, USA). After overnight incubation with the primary antibody, the membrane was incubated with the corresponding secondary antibodies (HRP goat anti-mouse IgG, 1 : 5000; HRP goat anti-rabbit IgG, 1 : 6000; Proteintech, IL, USA) at 25°C for 90 min. Detection was performed using an enhanced chemiluminescence (ECL) system (Advansta, CA, USA). Relative protein expression levels were analyzed using the Quantity One v4.6.2 software.

### 2.9. Immunofluorescence (IF)

After rinsing with PBS three times, the cells were fixed with 4% paraformaldehyde (#28908, ThermoFisher, China) for 30 min. Next, the cells were infiltrated with 0.3% Triton X-100 for 30 min. After 60 min of blocking with 5% BSA, the cells were incubated overnight at 4°C with primary antibodies against BSA. The primary antibodies used for IF were *α*-SMA (1 : 50; BOSTER, CA, USA), vimentin (1 : 50; Proteintech, IL, USA), and VE-Cad (1 : 50; CST, MA, USA). The cells were subsequently incubated at 37°C for 90 min with the corresponding secondary antibodies: CoraLite594-conjugated goat anti-mouse IgG (H + L) (1 : 200; Proteintech, IL, USA) and CoraLite594-conjugated goat anti-rabbit IgG (H + L) (1 : 200; Proteintech, IL, USA). Cells were then stained with DAPI for 10 min. A Motic image system was used for the observation and acquisition of images. Fluorescence intensity was analyzed using ImageJ.

### 2.10. Dual-Luciferase Reporter Gene Assay

To determine the direct target of miR-450a-2-3p, 293 T cells that were cotransfected with miR-450a-2-3p mimic or NC-mimic and the 3′UTR of MAPK1 or the mutant 3′UTR of MAPK1 were cultured in 24-well plates for 48 h. The binding site of miR-450a-2-3p in the MAPK1 3′UTR was mutated, and the activity of luciferase from *Renilla reniformis* or *Photinus pyralis* was measured.

### 2.11. Cell Proliferation Assay

Cell Counting Kit-8 (Dojindo, Japan) was used to evaluate cell proliferation according to manufacturer's instructions. Briefly, CFs were inoculated into 96-well plates with recombinant human TGF-*β*1 and miR-450a-2-3p mimic or miR-450a-2-3p inhibitor and their respective controls. The cellular proliferation rate was measured at 4 h using a microplate spectrophotometer (Huisong, Shenzhen, China) at a wavelength of 450 nm.

### 2.12. Scratch Assay

Transfected HUVECs that proliferated to form a full layer were rinsed with sterile PBS. The cell scratch was made using a sterile pipette tip in the cell monolayer along the surface of the culture dish. Cell migration to the scratch area was measured immediately after creating a scratch and 6 h later. The cell area was quantified using ImageJ v1.53e (Media Cybernetics, Inc., Rockville, MD, USA). The migration rate of HUVECs was defined as migration area/original scratch area × 100%.

### 2.13. Statistical Analysis

Two-tailed Student's *t*-test was used to compare the two different conditions. In cases in which the data did not follow a normal distribution, the Mann–Whitney test was used. The experimental comparisons of three or more experimental groups were performed using Tukey's multicomparison test or one-way ANOVA with Dunnett's posthoc test. Correlation between indicators was analyzed using Pearson. All data were expressed as the mean ± SEM. GraphPad Prism 8.0 software (GraphPad Software, San Diego, CA, USA) was used for statistical analysis. *P* < 0.05 was considered statistically significant.

## 3. Results

### 3.1. Potential Functional Significance of LINC00636/miR-450a-2-3p/MAPK1 in AF

**Figure 1:**
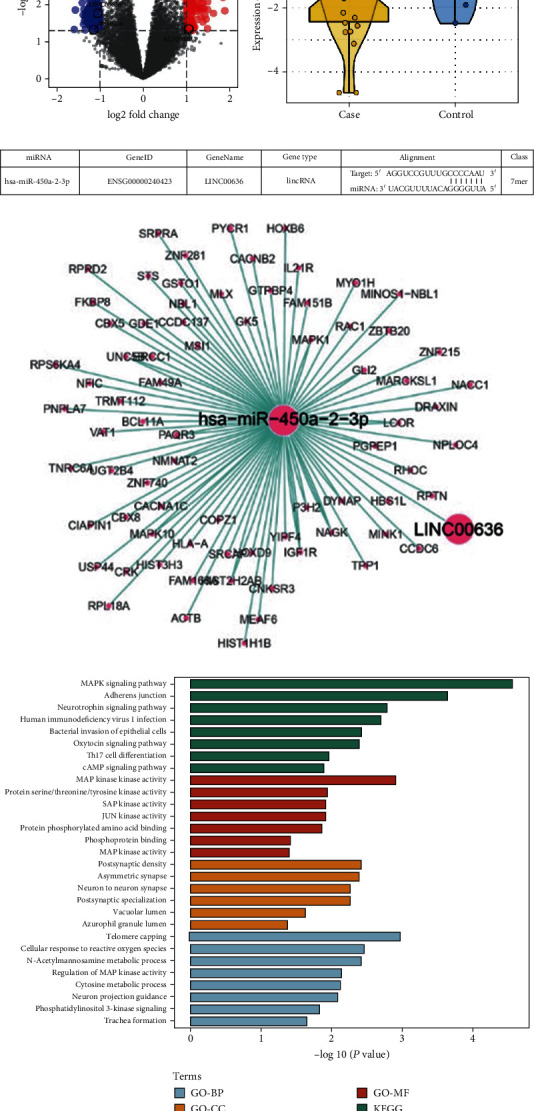
LncRNAs and their potential target genes in AF and SR patients. (a) Volcano plot of differentially expressed lncRNAs. Red and blue dots represent the high and low differential expression of lncRNAs, respectively. (b) Box plot of the LINC00636 expression in the two groups. (c) Map of binding sites between LINC00636 and miR-450a-2-3p. (d) Network map for LINC00636-related genes and miR-450a-2-3p. (e) GO and KEGG pathway enrichment analyses.

We performed genetic screening using the GSE55296 dataset, which consists of hundreds of lncRNAs with markedly different expression levels between the two groups as shown in the volcano plot (Figure 1(a)), among which the lncRNAs, namely, AC022532.1, LINC00636, and SNHG16, were downregulated (Figure 1(b)). According to Lincbase bioinformatic prediction, miR-450a-2-3p is a potential target gene of LINC00636 (Figure 1(c)). To preliminarily explore the potential functions of the dysregulated lncRNAs in AF, a functional enrichment analysis of the target genes of LINC00636 was performed based on GO terms. The mRNA targets of miR-450a-2-3p included a variety of genes, such as apoptosis-related genes MAPK1, BCL11A, and CBX8 (Figure 1(d)). Similar results were observed in the GO analysis of LINC00636. GO functional annotation was performed on the potential target genes at the three levels: biological process (BP), cellular component (CC), and molecular function (MF). The cAMP signaling pathway, Th17 cell differentiation, and MAPK1 were found to be closely related to these genes (Figure 1(e)).

### 3.2. Verification of the lncRNA Expression in Clinical Samples

**Figure 2:**
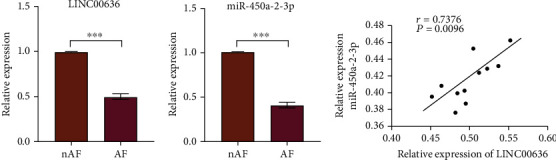
The expression of RNAs in human pericardial exosomes. (a, b) The expression levels of LINC00636 and miR-450a-2-3p determined using RT-qPCR. (c) LINC00636 expression levels are positively correlated with those of miR-450a-2-3p. Data is expressed as mean ± standard deviation calculated from three independent experiments. ^∗∗∗^*P* < 0.001.

To confirm the reliability of HTS results, exosomes derived from the PF of 12 nAF subjects and 12 AF patients were collected for RT-qPCR validation of LINC00636 and miR-450a-2-3p expression levels. As shown in Figure 2, the relative expression of LINC00636 in PF exosomes was reduced in the AF group compared to that in the nAF group, which was consistent with the HTS results. The expression of miR-450a-2-3p in the AF group was also lower than that in the nAF group. According to Pearson's analysis, the LINC00636 expression level was positively correlated with that of miR-450a-2-3p, which suggests a positive effect on AF.

### 3.3. Isolation and Characterization of Exosomes Derived from the PF and the Uptake of Exosomes by CFs

**Figure 3:**
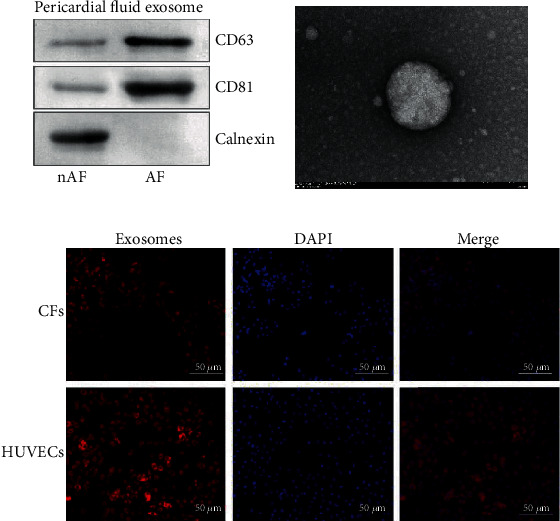
Characterization and the uptake of exosomes. (a) Biological markers of PF-derived exosomes were detected by western blot. (b) Images of exosomes acquired by TEM. (c) Uptake of PF-derived exosomes in CFs and HUVECs.

We hypothesized that lncRNAs carried by PF-derived exosomes may play a partial role in cardiomyocyte fibrosis. Therefore, we first isolated exosomes in the PF of the nAF and AF groups and confirmed their identity by analyzing the protein levels of several exosome markers using western blot (Figure 3(a)). A diagram of the isolated exosomes is shown in Figure 3(b). CFs and HUVECs stained with DAPI solution exhibited efficient uptake of exosomes as indicated by the internalization of PKH26-labeled exosomes (Figure 3(c)).

### 3.4. I LINC00636 Containing Exosomes Regulates miR-450a-2-3p and Affects CFs

**Figure 4:**
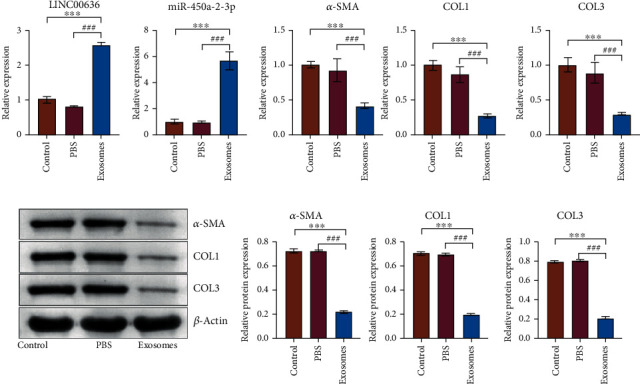
Exosomes containing LINC00636 could regulate miR-450a-2-3p and affects CFs. (a) Gene expression levels were detected by RT-qPCR. (b) *α*-SMA, COL1, and COL3 protein levels were detected by western blot. ^∗∗∗^ indicates significant differences with the control group, *P* < 0.001; ### indicates significant differences with the PBS group, *P* < 0.001.

To explore the effect of exosomes on CFs, we cocultured CFs and exosomes isolated from the PF of nAF patients. The expression level of LINC00636 in CFs was determined using RT-qPCR. Compared with the control and PBS groups, LINC00636 was highly expressed in the cells that had been incubated exosomes. Furthermore, miR-450a-2-3p was highly expressed in cells incubated with exosomes in than in the control group. LINC00636 may mediate miR-450a-2-3p expression levels, impacting the degree of fibrosis in CFs. Further, the expression levels of *α*-SMA, COL1, and COL3 were decreased at mRNA and protein levels (Figure 4). These results further suggest that exosomal LINC00636 may mediate miR-450a-2-3p expression to affect the degree of fibrosis in CFs.

### 3.5. MiR-450a-2-3p Inhibits TGF-*β*1-Induced Activation of CFs

**Figure 5:**
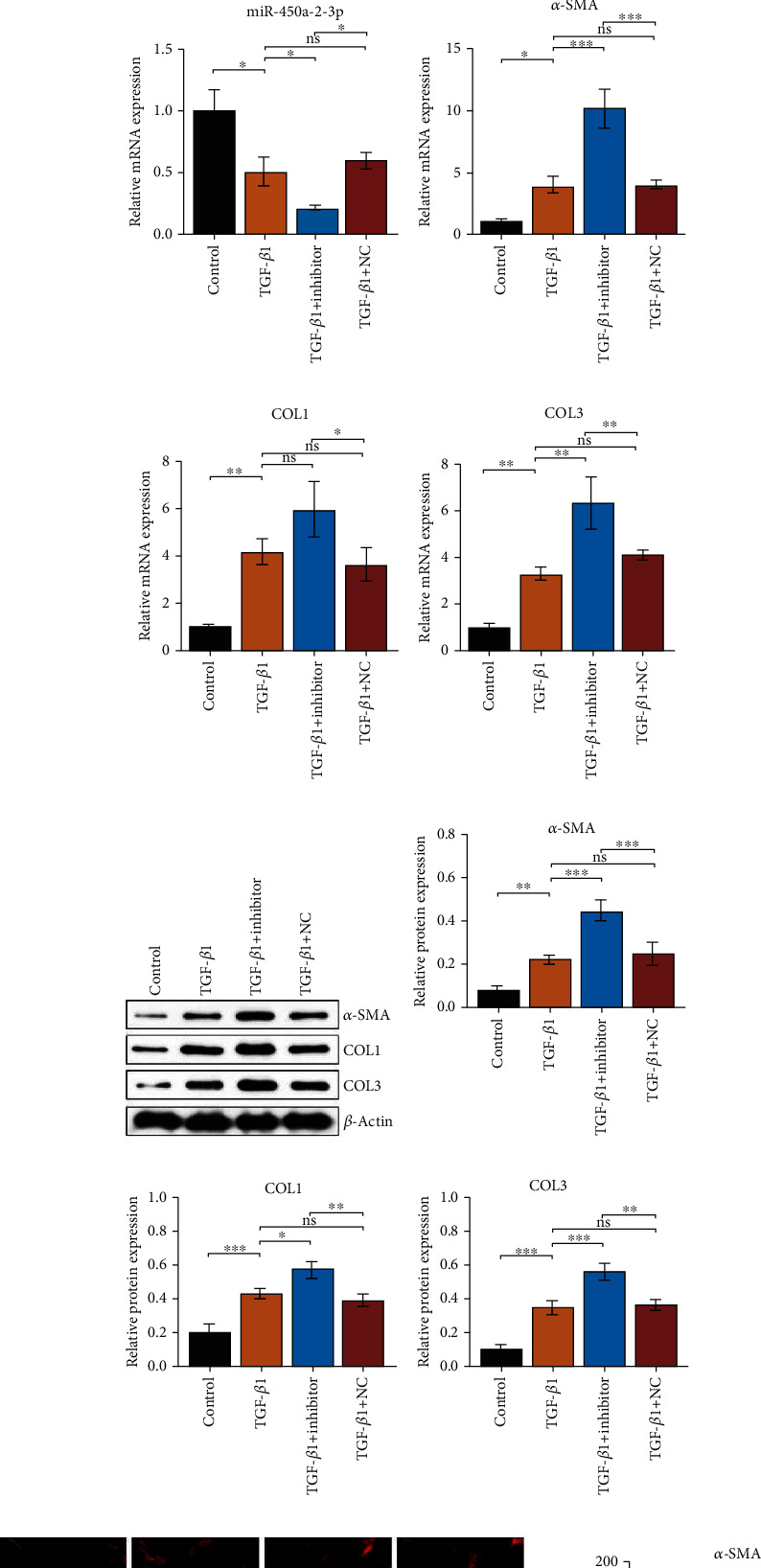
miR-450a-2-3p decreases TGF-*β*-induced activation and proliferation of CFs. miR-450a-2-3p, *α*-SMA, COL1, and COL3 mRNA expression in CFs transfected with mimics (a)–(d) and inhibitor (i)–(l) was detected using RT-qPCR. The protein levels of *α*-SMA, COL1, and COL3 in CFs transfected with mimics (e)–(h) and inhibitor (m)–(p) were determined using western blotting. The expression level of the miRNAs analyzed was normalized to the expression level of U6, and the expression level of mRNAs and proteins was normalized to the expression level of *β*-actin. Representative images of immunofluorescence staining for *α*-SMA in CFs transfected with mimics and inhibitor (r) (200× magnification) showed the antifibrotic effect of miR-450a-2-3p. CF proliferation measured by the CCK-8 assay after mimics (s) and inhibitor (t) treatment. Data is presented as the mean ± SEM of three independent experiments. ^∗^*P* < 0.05, ^∗∗^*P* < 0.01, ^∗∗∗^*P* < 0.001, ^∗∗∗∗^*P* < 0.0001.

To investigate the role of miR-450a-2-3p in the activation of CFs, TGF-*β*1-induced CFs were transfected with miR-450a-2-3p-mimic, control mimic, miR-450a-2-3p inhibitor, and control inhibitor. We measured the expression level of miR-450a-2-3p in untreated CFs and TGF-*β*1-treated CFs by RT-qPCR. Compared with the control condition, the miR-450a-2-3p expression was significantly downregulated in CFs treated with TGF-*β*. After transfection, the level of miR-450a-2-3p was higher in the mimic condition and lower in the inhibitor condition than in the TGF-*β*1-treated CFs, confirming that the transfection of mimic and inhibitor was successful (Figures 5(a) and 5(i)). The overexpression of miR-450a-2-3p attenuated TGF-*β*1-induced upregulation of *α*-SMA, COL1, and COL3 at transcription (Figures 5(b)–5(d)) and protein (Figures 5(e)–5(h)) levels. Conversely, the expression of *α*-SMA, COL1, and COL3 in TGF-*β*1-induced CFs was further promoted when the miR-450a-2-3p expression was inhibited at both mRNA and protein levels (Figures 5(j)–5(p)). Similarly, the IF results demonstrated that the miR-450a-2-3p mimic significantly inhibited the *α*-SMA overexpression in TGF-*β*1-induced CFs (Figure 5(q)), whereas the opposite was observed in the miR-450a-2-3p inhibitor group (Figure 5(r)). Based on the CCK8 assay, TGF-*β*1 stimulation promoted the proliferation of CFs, but this effect was reversed by miR-450a-2-3p mimic treatment (Figure 5(s)). In contrast, when transfected with miR-450a-2-3p inhibitor, proliferation of TGF-*β*1-induced CFs was further increased (Figure 5(t)). Together, these results suggest that miR-450a-2-3p inhibits the TGF-*β*1-induced activation of CFs.

### 3.6. MiR-450a-2-3p Downregulates the TGF-*β*1-Induced EMT of HUVECs

**Figure 6:**
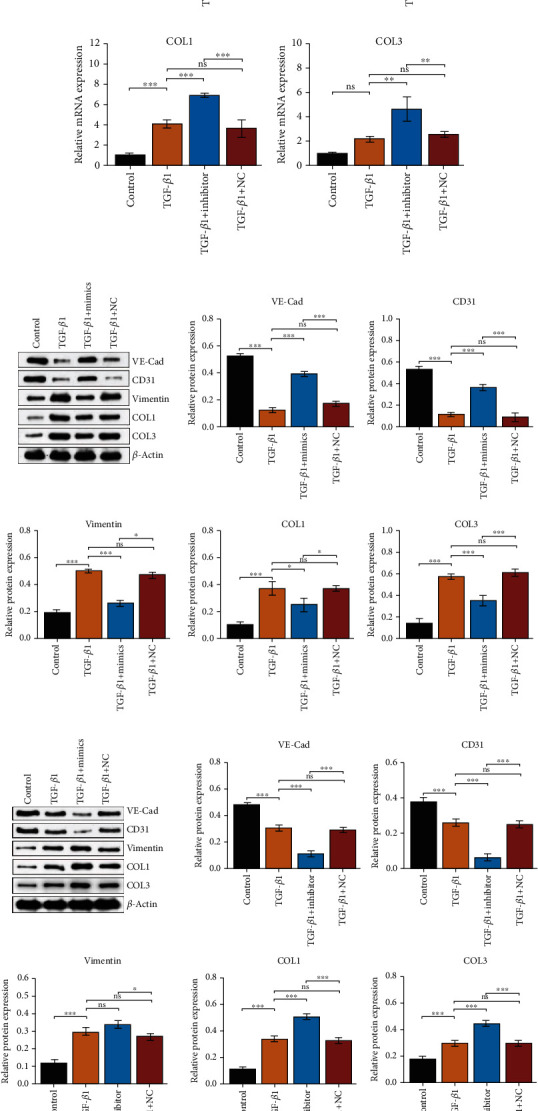
miR-450a-2-3p inhibits TGF-*β*1-induced EMT of HUVECs induced by TGF-*β*1. miR-450a-2-3p, VE-Cad, CD31, vimentin, COL1, and COL3 mRNA levels in HUVECs transfected with mimics (a)–(f) and inhibitor (g)–(l) were determined via RT-qPCR. The protein levels of VE-Cad, CD31, vimentin, COL1, and COL3 in CFs transfected with mimics (m) and inhibitor (n) were examined using western blot. Expression levels of miRNAs were normalized to that of U6, and the expression levels of mRNAs and proteins were normalized to that of actin. Representative images of VE-Cad immunofluorescence staining in HUVECs transfected with mimics (o) and inhibitor (p) (×200 magnification) showed the antifibrotic effect of miR-450a-2-3p. Representative images of the scratch assay of mimic- (q) and inhibitor-transfected (r) HUVECs (×100 magnification). Data is presented as the mean ± SEM of three independent experiments. ^∗^*P* < 0.05, ^∗∗^*P* < 0.01, ^∗∗∗^*P* < 0.001, ^∗∗∗∗^*P* < 0.0001.

To investigate whether miR-450a-2-3p can continuously inhibit EMT in HUVECs, we transfected TGF-*β*1-induced HUVECs with miR-450a-2-3p mimic, miR-450a-2-3p inhibitor, and their negative control and detected miR-450a-2-3p expression levels in HUVECs from the different treatment groups. TGF-*β*1 stimulation significantly reduced the expression of miR-450a-2-3p. However, this inhibition was partially abolished by the miR-450a-2-3p mimic, whereas it was intensified by the miR-450a-2-3p inhibitor (Figure 6(a)).

The protein expression levels of VE-CAD and PECAM-1/CD31 were decreased when the cells were stimulated with TGF-*β*1, whereas the expression levels of vimentin, COL1, and COL3 increased. MiR-450a-2-3p mimics reversed the changes observed in the expression levels of these genes (Figures 6(b)–6(g)). In the miR-450a-2-3p inhibitor group, the expression levels of VE-Cad and PECAM-1/CD31 were inhibited, and the expression of vimentin, COL1, and COL3 was significantly enhanced (Figures 6(h)–6(l)). The changes in the expression level of these proteins were consistent with the changes detected at the mRNA level (Figures 6(m) and 6(n)).

The immunofluorescence assay of VE-Cad further validated these results (Figures 6(o) and 6(p)). Enhanced endothelial cell migration is one of the characteristics of EMT. Scratch experiments showed that TGF-*β*1 accelerated HUVEC migration, an effect that was blocked by the miR-450a-2-3p mimic (Figure 6(q)), whereas miR-450a-2-3p inhibitor substantially facilitated HUVEC migration (Figure 6(r)). These results suggested that miR-450a-2-3p plays an antifibrotic role by suppressing EMT in HUVECs.

### 3.7. MAPK1 Is the Target of miR-450a-2-3p

**Figure 7:**
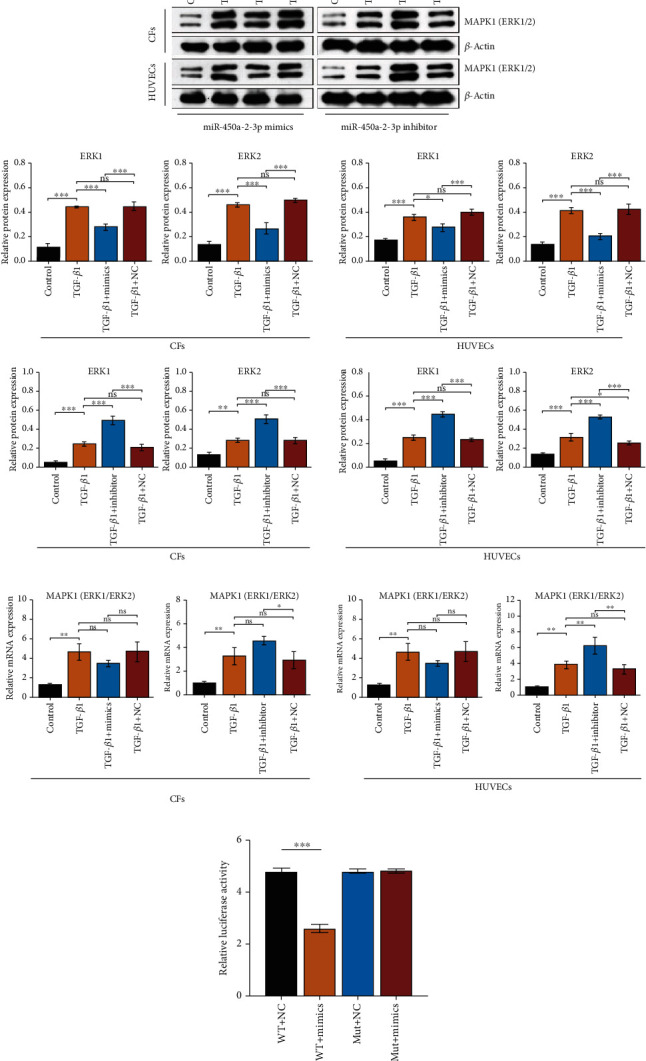
miR-450a-2-3p targets ERK1/2 (MAPK1). Protein (a) and mRNA (b) levels of ERK1/2 in CFs and HUVECS transfected with mimics or inhibitor were measured using western blot or RT-qPCR, respectively. The expression levels of mRNA and proteins were normalized to that of actin. (c) Relative luciferase activity of WT or mutant MAPK1 3′-UTR reporter cotransfected with miR-450a-2-3p mimics. Data is presented as the mean ± SEM of three independent experiments. ^∗^*P* < 0.05, ^∗∗^*P* < 0.01, ^∗∗∗^*P* < 0.001, ^∗∗∗∗^*P* < 0.0001.

To investigate the underlying mechanism of miR-450a-2-3p in CFs and HUVECs during cardiac remodeling, we assessed the mRNA and protein levels of endogenous MAPK1 in CFs and HUVECs. Transfection with miR-450a-2-3p-mimics significantly reduced the endogenous MAPK1 expression at both mRNA and protein levels, whereas transfection with miR-450a-2-3p inhibitor markedly augmented MAPK1 mRNA and protein expression levels (Figures 7(a) and 7(b)).

To further verify whether there is a targeting relationship between miR-450a-2-3p and MAPK1, luciferase reporter plasmids with the 3′UTR or 3′UTR-mut of MAPK1 were constructed. Cotransfection with miR-450a-2-3p and the wild-type 3′UTR of MAPK1 dramatically inhibited relative luciferase activity. Furthermore, transfection with miR-450a-2-3p had little influence on the relative luciferase activity of the mutant MAPK1 3′UTR (Figure 7(c)). These results demonstrate that MAPK1 is a direct target of miR-450a-2-3p.

### 3.8. Overexpression of MAPK1 Neutralizes the Effect of miR-450a-2-3p

**Figure 8:**
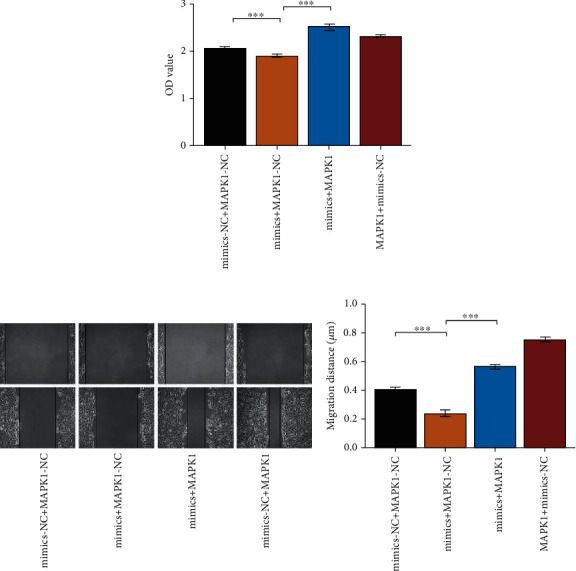
MAPK1 neutralizes the antifibrogenic effect by miR-450a-2-3p. (a) The proliferation of CFs was quantified by the CCK8 assay. CFs were transfected with mixture of miR-450a-2-3p mimics or NC (10 *μ*L) and MAPK1 overexpression plasmid or empty vector (2.5 *μ*g) for 48 h. (b) The scratch test was used to characterize the EMT in HUVECs. HUVECs were treated with blend of miR-450a-2-3p mimics or NC (10 *μ*L) and MAPK1 overexpression plasmid or empty vector (2.5 *μ*g) for 24 h. Data is presented as the mean ± SEM of three independent experiments. ^∗^*P* < 0.05, ^∗∗^*P* < 0.01, ^∗∗∗^*P* < 0.001, ^∗∗∗∗^*P* < 0.0001.

We next investigated whether MAPK1 is required for the miR-450a-2-3p activity. CCK8 data showed that the overexpression of MAPK1 eliminated the inhibitory effect of miR-450a-2-3p on CF (Figure 8(a)). In addition, the scratch assay showed that the inhibitory effect of miR-450a-2-3p mimic on HUVEC migration was markedly abolished by MAPK1 (Figure 8(b)). These findings suggest that miR-450a-2-3p is dependent on MAPK1 to regulate CF proliferation and EMT in HUVECs.

## 4. Discussion

Atrial structural remodeling is closely associated with cardiac fibrosis, which is considered to be fundamental to the occurrence and progression of AF. The abnormal expression of the ECM in CFs, the most common cell type in the heart, plays a deleterious role in cardiac fibrosis [15]. So far, there are still only a few effective therapeutic strategies for cardiac fibrosis due to the great difficulty of reversing cardiac fibrosis [16]. Hence, researching potential targets to inhibit myocardial fibrosis is essential to develop new strategies to prevent and cure atrial fibrillation.

To date, an overwhelming majority of lncRNAs have not been well characterized. However, lncRNAs have been shown to be involved in almost every facet of gene regulation, including epigenetic regulation, imprinting, nuclear and cytoplasmic trafficking, transcription, and mRNA splicing. Thus, lncRNAs are involved in many diverse biological processes, including cell cycle, cell proliferation, apoptosis, and differentiation [10]. After we discovered the presence of LINC00636 in pericardial fluid, we further found that LINC00636 could mediate the effect of miR-450a-2-3p on CFs. We have not verified whether there is a direct targeted regulation of the two ncRNAs. In recent years, studies have shown that lncRNAs can adsorb miRNAs. Since there are no lncRNAs that target the promotion of miRNA expression, we speculate that there may be an unknown pathway between LINC00636 and miR-450a-2-3p, which we seek to investigate to in our future research.

miRNAs, which are endogenous small noncoding RNAs, play important roles in regulating cardiac remodeling. For example, miR-30/133 regulates myocardial fibrosis by suppressing the expression of connective tissue growth factor in left ventricular hypertrophy [17]. In this study, the miR-450a-2-3p expression in CFs and HUVECs was downregulated under the induction of TGF-*β*1, which may be an important factor in the progression of fibrosis. However, when the miR-450a-2-3p expression was upregulated, the TGF-*β*1-induced overexpression of COL1, COL3, and *α*-SMA was inhibited, suggesting the inhibition of CF activation. On the other hand, when miR-450a-2-3p was upregulated in TGF-*β*1-stimulated HUVECs, VE-Cad, PECAM-1/CD31, vimentin, COL1, and COL3 were upregulated, indicating that miR-450a-2-3p suppressed TGF-*β*1-induced EMT in HUVECs. TGF-*β*1 is one of the most effective factors in inducing cardiac fibrosis among various regulatory stimuli of AF [18, 19]. TGF-*β*1 signaling involves at least two independent pathways, typical Smad-dependent pathway [20] and Smad-independent or certain atypical pathways [21, 22]. Studies have shown that endothelin 1 (ET-1) and TGF-*β*1 jointly promote myofibroblast differentiation [23]. This may be related to the ability of ET-1 to induce ECM production in fibroblasts and differentiation of myofibroblasts [24]. Similarly, AngII induces the production of ET-1 through MAPK1 and reactive oxygen species, thereby promoting fibroblast activation and fibrosis [25]. Additionally, TGF-*β*1-induced production of MAPK1 is required for the expression of CTGF, which is a key marker of myofibroblast differentiation [26, 27]. In essence, the TGF-*β*1/MAPK1 pathway may play a key role in atrial fibrosis [28].

MAPK1, a member of the MAPK family, has been reported to be involved in a variety of biological processes, such as myocardial fibrosis [22–30]. In an AF dog model, TRPC3 was found to regulate the proliferation of myocardial fibrosis by affecting Ca^2+^ influx through the MAPK1/miRNA-26/NFAT pathway, thereby increasing the expression of TRPC3 in the myocardium [30]. Some miRNAs play a role in pathological processes by regulating MAPK1. For instance, Thum et al. found that miR-21 regulates the MAPK1 signaling pathway, affecting the growth of CFs and the secretion of related cytokines, thereby affecting the process of interstitial fibrosis [29]. We confirmed the relationship between miR-450a-2-3p and MAPK1 through bioinformatic prediction analyses and dual luciferase assay. Furthermore, CCK8 and scratch assays showed that the MAPK1 overexpression could counteract the effect of miR-450a-2-3p. These results suggested that both the effects of miR-450a-2-3p on CF proliferation and EMT in HUVECs were affected by MAPK1.

In conclusion, exosomal LINC00636 may mediate the effect of miR-450a-2-3p through the MAPK1 pathway on the viability of CFs, which might be used to treat myocardial fibrosis. Exosomes containing LINC00636 in human pericardial fluid promoted the expression of miR-450a-2-3p to inhibit MAPK1 and improve cardiac fibrosis in patients with atrial fibrillation.

## Data Availability

The original data is GSE55296 of the GEO database.

## Supplementary Materials

Supplementary Materials All primer sequences were used in the study.
