# Supplementary material for: Exosomes Containing LINC00636 Inhibit MAPK1 through the miR-450a-2-3p Overexpression in Human Pericardial Fluid and Improve Cardiac Fibrosis in Patients with Atrial Fibrillation
Source: Mediators Inflamm. 2021 Jun 28;2021:9960241. doi: 10.1155/2021/9960241 (PMC8257384; doi:10.1155/2021/9960241)
Supplement: Supplementary Materials — All primer sequences were used in the study. [file 9960241.f1.docx]

Table1 All primer sequences were used in the study

| Gene | Sequences (5'-3') |
| --- | --- |
| LINC00636 | F: CATTTCCAGGTCCGTTTGC |
|  | R: AATTTAGCCACGTTATCCCAA |
| H-VIM | F: CCCTTGACATTGAGATTGCCACC |
|  | R: ACCGTCTTAATCAGAAGTGTCCT |
| H-VE-cad | F: ATGAGATCGTGGTGGAAGCG |
|  | R: ATGTGTACTTGGTCTGGGTGA |
| H-CD31 | F: CCACGCCTAGCCAAAATCAC |
|  | R: CATGTGGCCCCTCAGAAGAC |
| M-COL3A1 | F: ACGTAAGCACTGGTGGACAG |
|  | R: CAGGAGGGCCATAGCTGAAC |
| H-COL3A1 | F: CGCCCTCCTAATGGTCAAGG |
|  | R: TTCTGAGGACCAGTAGGGCA |
| M- COL1A1 | F: CTGGTGCTCGCGGTAACGAT |
|  | R: CAGCACCAGGGTTTCCAGCA |
| H-COL1A1 | F: GCAAGAACCCCGCCCGCACC |
|  | R: GCTCTCGCCGAACCAGACATGCC |
| M-α-SMA | F: GCCCCTGAAGAGCATCCGAC |
|  | R: CCAGAGTCCAGCACAATACCAGT |
| H-α-SMA | F: CTATGAGGGCTATGCCTTGCC |
|  | R: GCTCAGCAGTAGTAACGAAGGA |
| H-MAPK1 | F: ATCCCCATCACAAGAAGACCTG |
|  | R: AGCCTGTTCTACTTCAATCCTCT |
| M-MAPK1 | F: GGTTGTTCCCAAATGCTGACT |
|  | R: CAACTTCAATCCTCTTGTGAGGG |
| miR-450a-2-3p | F: GTACGAATTGGGGATGCTTTG |
|  | R: GGTGGGGACATTTTGCATTCAT |
| U6 | F: CTCGCTTCGGCAGCACA |
|  | R: AACGCTTCACGAATTTGCGT |
| β-actin | F:ACCCTGAAGTACCCCATCGAG |
|  | R: AGCACAGCCTGGATAGCAAC |
